# Assessing The Spatial Dependence of Adaptive Loci in 43 European and Western Asian Goat Breeds Using AFLP Markers

**DOI:** 10.1371/journal.pone.0086668

**Published:** 2014-01-30

**Authors:** Licia Colli, Stéphane Joost, Riccardo Negrini, Letizia Nicoloso, Paola Crepaldi, Paolo Ajmone-Marsan, the ECONOGENE Consortium

**Affiliations:** 1 Istituto di Zootecnica, Laboratorio di Genetica Animale, Università Cattolica del Sacro Cuore di Piacenza, Piacenza, Italy; 2 BioDNA Research Center, Università Cattolica del Sacro Cuore di Piacenza, Piacenza, Italy; 3 Laboratory of Geographic Information Systems (LASIG), School of Architecture, Civil and Environmental Engineering (ENAC), Ecole Polytechnique Fédérale de Lausanne (EPFL), Lausanne, Switzerland; 4 Associazione Italiana Allevatori, Roma, Italy; 5 Dipartimento di Scienze Veterinarie e Sanità Pubblica, Università degli Studi di Milano, Milano, Italy; Auburn University, United States of America

## Abstract

**Background:**

During the past decades, neutral DNA markers have been extensively employed to study demography, population genetics and structure in livestock, but less interest has been devoted to the evaluation of livestock adaptive potential through the identification of genomic regions likely to be under natural selection.

**Methodology/Principal findings:**

Landscape genomics can greatly benefit the entire livestock system through the identification of genotypes better adapted to specific or extreme environmental conditions. Therefore we analyzed 101 AFLP markers in 43 European and Western Asian goat breeds both with Matsam software, based on a correlative approach (SAM), and with Mcheza and Bayescan, two F_ST_ based software able to detect markers carrying signatures of natural selection.

Matsam identified four loci possibly under natural selection – also confirmed by F_ST_-outlier methods – and significantly associated with environmental variables such as diurnal temperature range, frequency of precipitation, relative humidity and solar radiation.

**Conclusions/Significance:**

These results show that landscape genomics can provide useful information on the environmental factors affecting the adaptive potential of livestock living in specific climatic conditions. Besides adding conservation value to livestock genetic resources, this knowledge may lead to the development of novel molecular tools useful to preserve the adaptive potential of local breeds during genetic improvement programs, and to increase the adaptability of industrial breeds to changing environments.

## Introduction

Neutral DNA markers have been extensively employed, during the last decades, to infer population genetics parameters, population structure and demographic trends, both in wildlife and livestock species [1]–[3]. Much scientific interest is now focused on investigating adaptive genetic variation [4]–[5] and on identifying genomic regions likely to be under selection [6]–[9]. So far, several methods have been proposed [5]–[6], [10]–[13]: some are based on candidate gene approaches which test whether or not a specific locus is a true target of selection by means of a number of different statistical methods [14]–[15]; others are designed to identify chromosomal regions affecting the phenotypes of complex adaptive traits (e.g. disease resistance), by measuring the association between different genotypes and the phenotype of interest [16].

The population genomics approach [4] searches for selection signatures by analyzing the variation of genetic diversity parameters along chromosomes, to discriminate between genomic regions under locus-specific (selection) and genome-wide (genetic drift, inbreeding and migration) effects [17]. The major limitation of this approach, however, is that it is blind respect to the causative selection forces. Signatures of selection for adaptive traits can be partially targeted by properly designing the experiment (e.g. contrasting groups of breeds reared in different environmental conditions), but the disentanglement of the effects linked to specific environmental variables remains impossible.

The approach based on spatial analysis and multiple parallel logistic regressions [9] offers a different perspective. Based on the geographical coordinates of the sampling locations, a set of environmental variables describing these locations are regressed on the genetic profile of each individual in search for robust association. The association between alleles and climatic variables is suggestive of signatures of adaptation and gives information on the environmental forces acting on the genome. This approach was named landscape genomics [9], [18]–[19] and permits to seek genomic regions influencing the ability of animals to cope with climatic variations. It is fast and integrates spatial heterogeneity, but it also makes implicit assumptions that have to be taken into account, among which the main ones are that a) the functional relationship between the spatial distribution of alleles and the environmental variables is assumed to be constant (this might not be the case if the environment has changed over time), and that b) selection has had enough time to create a functional relationship between the allele distributions and the environmental variables [20]. However, landscape genomics can potentially benefit the entire livestock system through the identification of better adapted genotypes, hence more suitable to rear in specific environmental contexts. This, in turn, can significantly reduce the costs required to counter the consequences of climate changes on animal production systems.

So far, this type of analysis has been rarely applied to livestock breeds (sheep [9], goats [21], cattle [22]), while much research has been devoted to wild animal and plant species (see [18] and references therein). Most livestock genetics and genomics research is still focused on production traits and industrial breeds, while the characterization, the evaluation of genetic resources and the promotion of their sustainable use are receiving less attention, even if local breeds are a mean to provide income to a very large number of resource-poor smallholders.

The domestic goat is one of the five most important livestock species worldwide and plays an important role in the livelihood of a large proportion of small and marginal farmers and landless shepherds.

Goat genetic resources comprise 627 described breeds, distributed throughout the continents and representing a unique reservoir of genetic diversity [23]. Among these, about 400 are native to Asia (176) and Europe (195), including the Caucasus. Africa has the highest number of regional transboundary populations (15 out of 46 worldwide) followed by Europe (12) and Asia (11). About 40 breeds have been registered as international transboundary or cosmopolite.

The DAD-IS indicates (May 9, 2013) that the number of goat breeds in Europe is even higher, reporting 342 breeds in total, with an average of 8.1 per country. Notable outliers are Italy (54 native breeds), Germany (26), Spain (24) and Albania (20). Several other countries host more than 10 native breeds: Russian Federation (15), France, the Netherlands and Turkey (14), Austria (13), United Kingdom (12), Hungary and Switzerland (11).

The sustainability of goat farming in marginal and difficult areas is strongly dependent on local breeds, well adapted to specific agro-ecological conditions (e.g. harsh climate, tropical diseases, poor nutrition and beverage) and which represent, in both developed and developing countries, an invaluable source of meat, milk and skin [24]. At present, indiscriminate crossbreeding with cosmopolitan breeds and uncontrolled intermixing are threatening a number of local caprine genetic resources. In order to conserve the diversity of goat germplasm - now recognized as an issue of international concern – the characterization of local breeds molecular diversity and adaptive potential is urgently needed to serve as a rational basis for conservation strategies and sustainable breeding.

With this paper, our intention is to contribute to the understanding of the environmental forces acting on the goat genome, by applying both landscape genomic and F_ST_-outlier approaches to a set of presumably neutral AFLP markers genotyped in 43 European and Southwestern Asian goat breeds.

## Materials and Methods

### Ethics statement

All experimental procedures were approved by the European Commission, in accordance with the EU Directive 86/609. The EU Econogene contract was issued within FP5 (Framework Programme 5) in 2001. At that time within the EU there was no dedicated animal welfare/ethics committee to evaluate and approve issues specifically related to these aspects of research projects. The permission to carry out the sampling at each farm was obtained directly from the owners. Blood samples have been collected by veterinarians, complying with relevant national and international regulations and animal welfare requirements.

### Sampling and GPS data recording

Biological samples collection was carried out during years 2002 and 2003.

The sampling strategy consisted in a balanced effort of collecting information from a list of selected key breeds while ensuring spatial representativeness to prevent from over-representing any environmental condition. Thus peripheral blood samples were collected from 1239 animals belonging to 43 European and Southwestern Asian goat breeds (Table 1). Between 28 and 33 unrelated individuals were sampled in 10 farms covering the area of origin and the present distribution of breeds (Table 1; Figure S1). A maximum of 3 individuals per farm were sampled to reduce the relatedness among animals and to increase the breed representativeness. There are however exceptions in Turkey, where Hair (#43), Gurcu (#42) and Abaza goats (#40) were sampled in only two farms per breed; similarly, in Albania, Mati (#5) and Muzhake (#6) goats were collected in a single farm per breed, causing a slight overrepresentation of corresponding climatic conditions. Sex bias was minimized by sampling at least 1 male per farm. Geographical coordinates (longitude and latitude, decimal degrees, WGS84 geodetic reference system) of sampling locations, were either recorded with a GPS device with a dedicated protocol or derived from local maps.

**Table 1:** List of goat breeds included in the study (ID numbers on the first column was used to identify the breeds on the maps in Figures 5, 6 and Figure S1).

ID	Country	Acronym	Breed name	Sample size	% of polymorphic loci	Longitude	Latitude
1	Albania	ALCAP	CAPORE	30	63.5	20.622	40.878
2	Albania	ALDUK	DUKATI	17	62.7	19.501	40.290
3	Albania	ALHAS	HASI	23	58.8	20.450	42.203
4	Albania	ALLIQ	LIQENASI	30	58.7	20.739	40.691
5	Albania	ALMAT	MATI	27	58.7	20.004	41.865
6	Albania	ALMUZ	MUZHAKE	26	56.7	20.207	40.093
7	Austria	AUPIZ	PINZGAUER	30	62.5	12.771	47.351
8	Austria	AUTAS	TAUERNSCHECKEN	30	52.9	13.113	47.274
9	Switzerland	CHALP	SWISS ALPINE	56	57.7	8.220	46.723
10	Switzerland	CHGRS	GRISONS STRIPED	28	56.7	8.414	46.771
11	Switzerland	CHPCG	PEACOCK GOAT	29	55.8	8.530	47.066
12	Switzerland	CHSGB	ST. GALLEN BOOTED GOAT	29	54.8	7.926	46.880
13	Switzerland	CHVBN	VALAIS BLACK NECK	31	54.8	8.364	46.762
14	Germany	DEBDE	GERMAN ALPINE	29	56.3	9.076	49.871
15	Germany	DETWZ	THURINGIAN FOREST GOAT	28	52.9	10.314	50.842
16	France	FRALP	FRENCH ALPINE	42	61.5	5.638	45.188
17	France	FRCOR	CORSICAN	29	57.7	9.208	42.141
18	France	FRPYR	PYRENEAN	31	52.9	1.019	43.341
19	France	FRROV	ROVE	31	58.7	4.992	44.207
20	Greece	GRGRG	GREEK GOAT	30	58.7	24.037	38.859
21	Greece	GRSKO	SKOPELOS	31	56.7	23.307	39.330
22	Hungary	HUNAT	HUNGARIAN NATIVE	27	50.0	19.900	47.023
23	Italy	ITARG	ARGENTATA DELL'ETNA	31	64.4	14.995	37.991
24	Italy	ITBIO	BIONDA DELL'ADAMELLO	27	56.7	10.302	45.970
25	Italy	ITCAM	CAMOSCIATA DELLE ALPI	29	56.7	9.819	46.015
26	Italy	ITGIR	GIRGENTANA	31	51.0	13.872	37.506
27	Italy	ITGMO	GRIGIA MOLISANA	31	57.3	14.397	41.518
28	Italy	ITORO	OROBICA	30	55.8	9.562	45.878
29	Italy	ITSAR	SARDA	30	63.5	9.156	39.552
30	Italy	ITVAL	VALDOSTANA	31	55.8	7.430	45.708
31	Jordan	JOBAL	BALADI	19	64.4	35.643	31.324
32	Poland	PLBUK	POLISH FAWN COLOURED GOAT	30	53.4	18.201	52.470
33	Portugal	PTBRA	BRAVA	19	57.3	−8.010	41.559
34	Romania	ROCAR	CARPATHIAN	23	68.9	23.239	46.528
35	Spain	SPFLR	FLORIDA	27	53.4	−5.160	37.825
36	Spain	SPGDR	CABRA DEL GUADARRAMA	29	59.6	−4.107	40.555
37	Spain	SPMLG	MALAGUENA	26	60.6	−4.363	36.865
38	Spain	SPPYY	PAYOYA	28	55.3	−5.394	36.840
39	Spain	SPVRT	VERATA	26	60.2	−5.641	40.110
40	Turkey	TKABA	ABAZA	25	56.3	42.899	40.946
41	Turkey	TKANG	ANGORA	29	59.6	32.154	39.626
42	Turkey	TKGUR	GURCU	25	61.2	43.240	40.380
43	Turkey	TKHAI	HAIR	29	67.3	38.421	38.744

### Environmental variables

Altitudinal and climatic information was used to characterize the natural environment at each sampling site. Altitude was measured in the field with either an altimeter, or a GPS device, whenever possible, or derived from SRTM30 NASA, http://www2.jpl.nasa.gov/srtm/. All data were validated with the 3 arc second (approximately 90 meters) digital elevation model (DEM) of the Shuttle Radar Topography Mission [25]. The use of the DEM turned out to be efficient both in detecting anomalies (wrong measures in the field) and in assigning coherent altitudes in case of missing data. As for climatic data, they were recorded according to a latitude/longitude grid with a spatial resolution of 10 minutes (equivalent to approximately 12 km at the latitude of Switzerland). In each cell of the grid we took into account 9 monthly variables, plus their yearly mean (Table 2), produced by the Climatic Research Unit in Norwich (CRU; http://www.cru.uea.ac.uk) to characterize continental regions from 1961 to 1990 [26].

**Table 2:** List of the environmental variables used in Matsam association analyses: 118 total variables = altitude+9 climatic variables ×13 periods (12 months+yearly mean).

Variable	Description
Altitude	Altitude computed with NASA SRTM30 Digital Elevation Model
DTR	Yearly mean and monthly values of mean diurnal temperature range in deg C
FRS	Yearly mean and monthly values of number of days with ground-frost
PR	Yearly mean and monthly values of precipitations in mm/month
PRCV	Yearly mean and monthly values of the coefficient of variation of monthly precipitation in percent
REH	Yearly mean and monthly values of relative humidity in percent
SUN	Yearly mean and monthly values of percent of maximum possible sunshine
TMP	Yearly mean and monthly values of mean temperature in deg C
WET	Yearly mean and monthly values of wet-days (number of days with >0.1 mm rain per month)
WND	Yearly mean and monthly values of wind speed in m/s, 10 meters above the ground

### DNA extraction and AFLP production

Genomic DNA has been extracted from frozen whole blood using a commercial kit (GenElute™ Mammalian Genomic DNA Miniprep Kit-Sigma). AFLP marker profiles were generated following a standardized protocol (modified from [27]. See File S1 for further details). Selective amplification was performed with three highly informative EcoRI/TaqI primer combinations (see Table S1 in File S1) selected according to levels of polymorphism and number of bands.

### Identification of loci possibly under selection

#### Correlative approach

The Spatial Analysis Method (SAM) described by Joost and colleagues [9] was used to compute association models between environmental variables and AFLP markers. As recently pointed out by Jones et al. [28], being dominant and loosely distributed on the genome, these markers have lower information content when compared to multiallelic (e.g. microsatellites) or more dense (e.g. SNP chip) marker panels, but at the time when the research was carried out, they represented the only tool available to perform a genome-wide scan of molecular variation in goat.

Association models were calculated with the software Matsam [29], which is based on spatial coincidence, one of the six spatial analysis concepts distinguished by Goodchild [30].

The association models relate the genetic profile of the individuals to environmental parameters measured at their geographical coordinates of origin. The method employs a geo-referenced data set describing individuals with environmental parameters and a binary matrix indicating the presence or the absence of AFLP bands. Univariate logistic regression analysis is used to determine the degree of association between the values of the environmental parameters and the frequencies of each AFLP phenotype. Then, the significance of the models generated by all possible pair-wise combinations of genetic marker versus environmental parameter is calculated. To this end, two statistical tests are used: the Wald and the Likelihood ratio statistics, integrating a correction for multiple hypothesis testing [9]. A model is considered significant only if both tests reject the corresponding null hypothesis. Markers included in statistically significant models are likely to host molecular variation involved in the processes of adaptation to the environment. In this study, we deliberately implemented univariate models only, as an exploratory step to detect the main environmental variables possibly selecting particular regions of the genome.

Finally, to validate the results produced by Matsam, the goat AFLP data were also analysed with two other population genomic approaches able to detect selection sweeps.

#### F_ST_-outlier methods

Mcheza [31] is a front-end program facilitating the use of Dfdist software [32]–[33], a modification of Fdist [32] which allows for dominant markers and implements the method of Zhivotovsky [34] to estimate allele frequencies. The Fdist method relies on the principle of genetic differentiation between populations, expressed in this case through the F_ST_ index [35]. In a simulation model of the infinite number of possible values for populations of constant size, with a migrant exchange rate at equilibrium (the infinite island model), the F_ST_ index introduced by Wright [36] corresponds to the probability that two alleles drawn at random from a population descend from an ancestral allele present in the same population [6]. The Fdist method is a variant of the Lewontin-Krakauer test [37], which relies on the idea that, in a given population, all genomic loci have comparable F_ST_ values because they share identical demographic histories. Only loci subject to active selection deviate from this rule [6].

The other software used was Bayescan version 2.1 [12], which exploits a Bayesian inference method to directly estimate the posterior probability (PP) for each locus to be under selection. The procedure of statistical inference implemented in Bayescan uses observations to update or to newly infer the probability that a hypothesis may be true. To identify loci possibly under selection, the software both provides Bayes factors and Posterior Odds (PO) scores. The latter, in particular, indicate how more probable a model with selection is compared to a neutral model by calculating the ratio between their PPs [38].

### Spatial statistics

Using the geographical coordinates of the farms, the spatial autocorrelation of the frequency of the markers detected as possibly under selection was measured with the help of Local Indicators of Spatial Association (LISA) [39]. LISA indicators are based on the statistical index I developed by Moran [40] and evaluate the existence of clusters in the spatial arrangement of a given variable due to underlying autocorrelation.

In this paper, we used bivariate LISA within a given spatial weighting scheme (see below), to identify and map local clusters of high or low marker frequencies (the first variable) significantly correlated with environmental variables (the second variable) possibly exerting selective pressures on them. This allowed to refine the identification of association signals detected by Matsam over the whole study area and to check whether these associations involved particular breeds only.

The study area is very large, and the spatial distribution and density of farms are heterogeneous (e.g. a dense distribution of sampling points in the Alps versus four sparse locations in Turkey). Since it was difficult to apply a weighting specific to each situation, the K = 90 nearest neighbours criterion was adopted. Indeed, due to our sampling scheme, on average 29 nearest neighbours have a high probability to belong to the same breed; in particular, there is a small area in Eastern Turkey with 50 animals belonging to Gurcu and Abaza breeds only, and another small area in Albania with 60 animals belonging to Liqenasi and Capore breeds only. Due to these occurrences, we set K to 90 to guarantee the inclusion of a representative number of animals from outside the local area.

## Results

### AFLP markers

The three AFLP primer combinations produced a total of 101 polymorphic bands (34 per primer combination on average). The percentages of polymorphic loci per breed ranged between 50.0% in HUNAT breed and 68.9% in ROCAR breed (Table 1).

The final dataset included 125139 data points, with a level of missing data of 0.50%, and is available from the authors upon direct request.

### Association models (Matsam)

**Figure 1:**
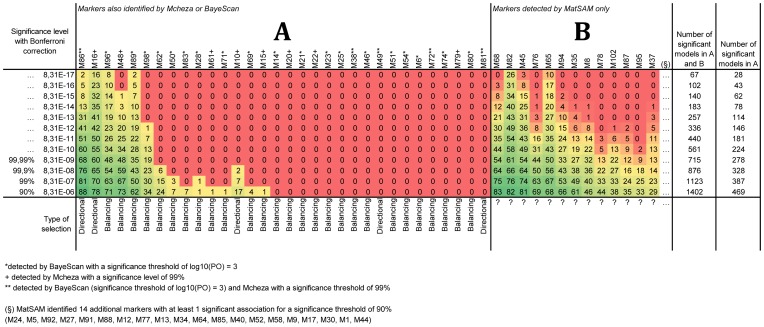
Number of significant models identified by MATSAM for the most significant confidence levels. Number of models identified by Matsam, Bayescan and Mcheza (panel A) and by Matsam alone (panel B) at different levels of statistical significance (Wald test). The colour shades vary from dark green = large number of significant associations, to red = no significant associations.

With a standard 99% confidence level, on a total of 11918 models computed (118 environmental parameters ×101 AFLP markers) Matsam identified 1123 significant associations (9.4%, Figure 1). When applying the most conservative confidence level (8.31E-17), 67 significant models (0.56% of the total) were shared between markers M16, M65, M82, M86, M89 and M96, with marker M82 showing the largest number (n = 26) of significant associations. The environmental variables involved were wind, diurnal temperature range, precipitation frequency, relative humidity and percentage of maximum possible sunshine. Marker M16 is significantly associated (16 models) with diurnal temperature range (dtrjun, dtrsep, dtroct, dtrnov, dtryear), frequency of precipitation (rdosep, rdooct, rdonov), relative humidity (rehsep, rehoct) and solar radiation (sunapr, sunjun, sunaug, sunsep, sunoct, sunyear). Marker M65 is associated (10 models) with precipitation, frequency of precipitation and solar radiation. Marker M96 is involved in 8 models showing association with ground frost, temperature and frequency of precipitation. Finally, marker M86 is significantly associated with relative humidity (1 model) and the percentage of maximum possible sunshine (1 model).

### F_ST_-outlier methods

With a confidence level set to 99%, Mcheza detected a total of 13 outlier loci possibly under selection, among which 5 under directional (M10, M16, M49, M81, and M86) and 8 under balancing selection (M15, M20, M22, M38, M48, M61, M72, and M79).

**Figure 2:**
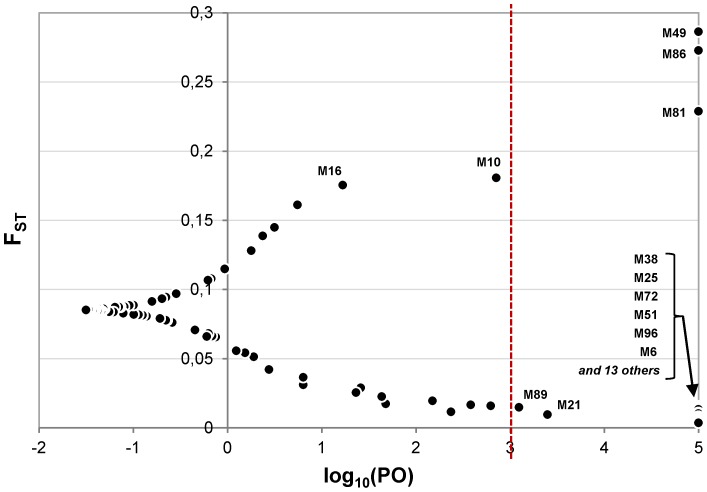
Outlier loci identified by BayeScan. Axis X shows the posterior odds (PO), i.e. the ratio between the posterior probability (PP) of the model with selection and the PP of the neutral model. The Y axis shows the F_ST_ index values.

In parallel, with a significance threshold set to log10(PO) = 3 (“Decisive evidence for selection” according to Jeffreys' scale of evidence) [41], BayeScan was able to identify a total of 24 outlier loci, among which 3 possibly under directional (M49, M81, M86; upper right corner in Figure 2), and 21 (M6, M14, M21, M23, M25, M28, M38, M46, M50, M51, M54, M62, M69, M71, M72, M74, M80, M83, M89, M96, and M98; lower right corner in Figure 2) possibly under balancing selection. A subset of five markers (M38, M49, M72, M81 and M86; Figure S2) were highlighted as outliers by both Mcheza and BayeScan.

### Combined approaches

With a significance level of 8.31E-15, among the most significant models identified by Matsam, five markers (M16, M48, M86, M89 and M96) were also detected as outliers by population genomics approaches (Figure 1, panel A). Among them, three (M16, M48 and M86) were detected by Mcheza (with a 99% confidence level) and three by BayeScan (M86, M89 and M96 with a PP of 1.0 and a significance threshold set to log10(PO) = 3). Only locus M86 was identified by all three methods (Figure S2).

**Figure 3:**
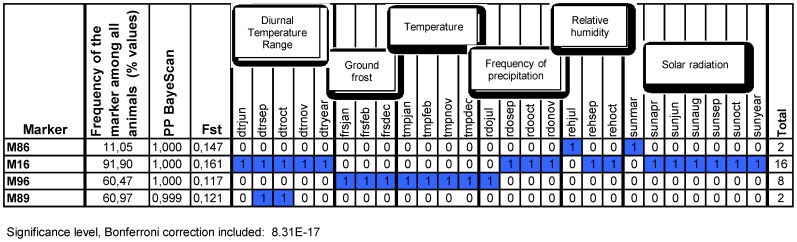
The four AFLP markers most significantly associated with environmental variables. From left to right: the average frequency of the marker over the whole study area; the Posterior Probability for the marker to be under selection provided by BayeScan; the F_ST_ value provided by Mcheza; the detail of monthly or yearly environmental variables associated with the corresponding marker.

When only the most conservative significance level of Matsam (8.31E-17) was considered, the number of models was reduced to 67 and we could, therefore, single out the environmental variables associated with the 4 highest ranking markers (M16, M86, M89 and M96; Figure 3).

## Discussion

### Signatures of natural selection and adaptation

**Figure 4:**
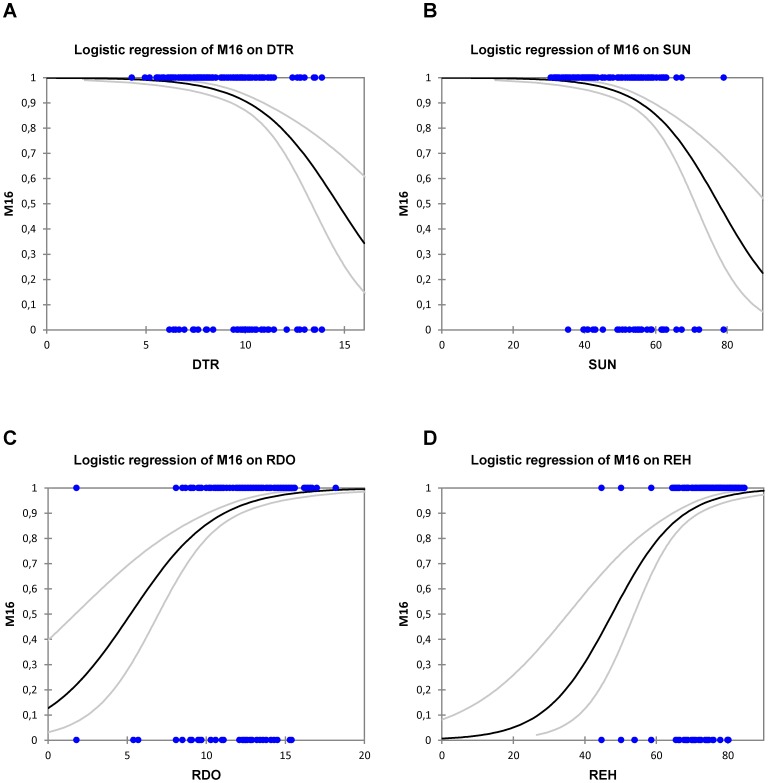
Logistic regression of marker M16 on four environmental variables. Results of the logistic regression of marker M16 on A) yearly mean of diurnal temperature range (DTR), B) yearly mean of the percentage of maximum possible sunshine (SUN), C) frequency of precipitation in November (RDO), and D) relative humidity in October (REH). Blue dots represent locations where the band is present (1) or absent (0). Grey lines show the upper and the lower limit of the confidence interval at 99.9%.

**Figure 5:**
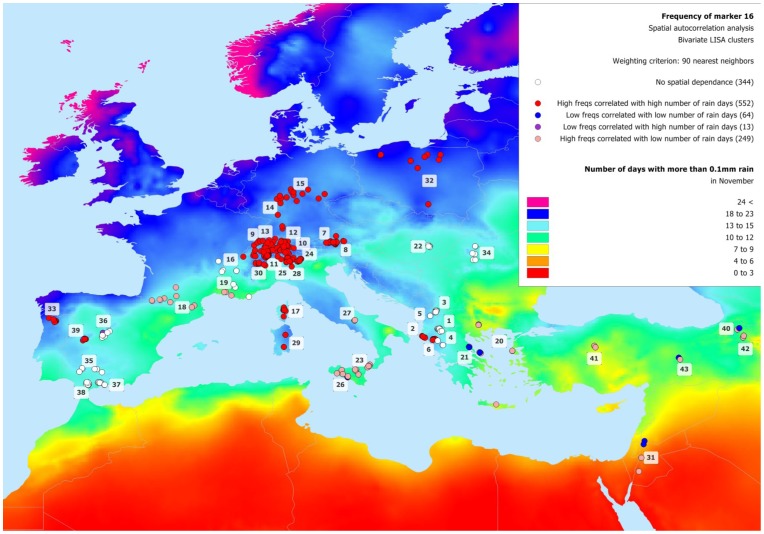
Clusters resulting from the bivariate LISA analysis of the frequency of marker M16. The plot shows the distribution of the clusters obtained from the bivariate LISA analysis of the correlation of the frequency of marker M16 with the weighted values of the environmental variable “number of days with more than 0.1 mm of rain in November”. The colors of the cluster correspond to different spatial autocorrelation regimes: red = high marker frequencies correlated with high mean of environmental variables values measured at the nearest 90 neighbouring farms (see the text for further details); blue = low marker frequency correlated with low environmental variable values; purple = low marker frequency correlated with high environmental variable values; pale red = high marker frequency correlated with low environmental variable values. Locations with frequencies showing no spatial dependence are displayed in white.

The analysis of the spatial distribution of the frequency of the four markers most significantly associated with environmental variables (Figures 1 and 3) highlights some interesting evidence. M16, identified to be under natural selection by Matsam and Mcheza, is present in more than 90% of the sampled animals, and is geographically distributed in a rather homogeneous way, with frequencies decreasing to about 0.6 on average in the easternmost part of the sampling range (Jordan and Turkey; Figure S3). Any environmental interpretation is made difficult because M16 is significantly associated with 4 different environmental variables (Figure 3): diurnal temperature range (DTR, 5 variables), the percentage of maximum possible sunshine (SUN, 6 variables), frequency of precipitation (RDO, 3 variables), and relative humidity (REH, in September and October). Figure 4 well shows that the frequency of marker M16 is negatively correlated with the yearly mean of DTR and with the yearly mean of SUN (panels A and B, respectively), while it is positively correlated with RDO in November and REH in October (panels C and D, respectively), highlighting a possible selective effect of the frequency of precipitation and humidity on this genomic region. By the way, the latter shows the best goodness-of-fit value among these models as translated by an Akaike Information Criterion (AIC) [42] of 594.5 (it is equal to 605.7, 607.9 and 613.6 for sunyear, dtryear and rehoct respectively). Joost and colleagues [9] obtained a similar result in sheep, where DYMS1 microsatellite marker - known to be involved in parasite resistance [43] - was found to be associated with the number of wet days, an environmental variable greatly influencing parasite load. Accordingly, in Figure 5, a large cluster of M16 high frequencies correlated with a high number of rain days in November is observed in the Alps and in Northern Europe. In this situation, the Moran's I measuring the global spatial autocorrelation over the whole study area is 0.26, while highlighting local regimes as evidenced by the different LISA clusters. Opposed to the North-Western “high-high” cluster mentioned above, the South-Eastern part of the study area shows several places (e.g. southern Greece, Jordan and Turkey; Figure 5) where correlation clusters with opposite behaviours coexist: either “low-low” (low marker frequencies correlated with low number of rain days) or “high-low” (high marker frequencies correlated with low number of rain days). It has to be noted that because of the superposition of many animals in Turkey, the map hides additional blue “low-low” and pale red “high-low” dots. For the Hair breed (#43), we actually have 10 blue dots and 19 pale red. For the Angora breed (#41), we have 9 blue dots and 20 pale red. For the Gurcu breed (#42), we have 9 blue dots and 16 pale red. And finally for the Abaza (#40), there are 10 blue dots and 15 pale red. Most of the area where M16 frequency shows this dual behaviour is close to the goat domestication centre [44]; possible explanations can be: i) a lack of directional selective pressure in this area due to different precipitations regimes alternating frequently during the year. Therefore, the present day “high-high” North-Eastern European cluster may have derived from a gradual selective pressure exerted by different precipitation regimes on the genomic region surrounding M16, during the migration of goats towards North-Eastern Europe along with humans; ii) in Anatolian and European populations the levels of Linkage Disequilibrium between M16 and the selected genomic region may differ, due to changes in population structure or effective population size, so that M16 has become linked to the selected gene after the spread out of the domestication centre.

**Figure 6:**
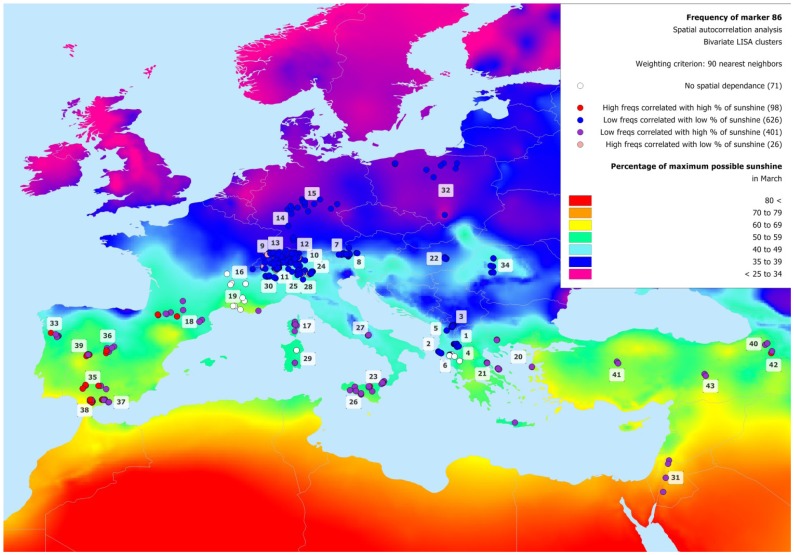
Clusters resulting from the bivariate LISA analysis of the frequency of marker M86. The plot shows the distribution of the clusters obtained from the bivariate LISA analysis of the correlation of the frequency of marker M86 compared with the weighted values of the environmental variable “percentage of maximum possible sunshine in March”. The colors of the cluster correspond to different spatial autocorrelation regimes, as explained in Figure 5.

M86, which resulted under selection according to all three methods, is present in only 11% of sampled goats (Figure 3) and shows a higher frequency in breeds located in the Iberian Peninsula and in the Pyrenees (Figure S3). This marker appears to be inversely correlated with REH in July and positively with the percentage of maximum possible sunshine in March (Moran's I = 0.23), as shown by the large cluster of M86 low frequencies correlated with a low percentage of maximum possible sunshine in the Alps (Figure 6). In this area, the few pale red exceptions are represented by French Alpine, Swiss Alpine, and Valais Black Neck breeds in which M86 is present (Figure S3). The Mediterranean islands and the South-Eastern part of the study area are characterized by a very low frequency of M86 associated with a high percentage of possible sunshine. Finally, the Iberian Peninsula and the Pyrenees exhibit a different picture with breeds showing a high M86 frequency in a sundrenched geographical region. Interestingly, the processing of the logistic regression between M86 and SUN (in March) restricted to individuals from the Iberian Peninsula and the Pyrenees provided a result worse than when calculated between all individuals in the data set. The partial model was significant with a 95% confidence level (and not with 99%), showed a pseudo R^2^ of 0.033 and an Area Under the Curve (AUC) of 0.59. In comparison, the global model shows a pseudo R^2^ of 0.13 with an AUC of 0.75. The details of the bivariate LISA cluster analysis reveal that on a total of 184 animals from the Iberian Peninsula and the Pyrenees, 77 have a high M86 frequency associated with high SUN values, while 107 show low M86 frequencies associated with high SUN values. Importantly, all breeds are represented in each cluster categories with a minimum of 3 individuals. This means that in this area the marker in question is spatially dependent on SUN (there is no neutral location), but the latter environmental variable until now either selected M86 only in part of the individuals, or the high SUN local values coincide by chance with a high frequency of this marker as a result of demographic events (e.g. genetic drift or introgression from Africa).

M89 and M96 were identified by Matsam and BayeScan as under selection. For these two markers, the signature of the environmental pressure is clear (Figure 3). They are both present in about the 60% of the animals in the dataset, and have a homogeneous spatial distribution (Figure S3). M89 is likely to be selected by high values of diurnal temperature range in autumn, while M96, which is less frequent in the breeds of the Iberian Peninsula and in the Pyrenees, is possibly selected by the number of days with ground frost in winter (positive correlation), and by temperature in winter (inverse correlation).

### Compared advantages and drawbacks of F_ST_-outlier methods and correlative approaches

Advantages and drawbacks of correlative and population genomics approaches have already been discussed [9], [18], [20]. Here we want to extend the discussion into the usefulness and relevance of the concurrent use of the two types of approaches. The comparison of the results produced by F_ST_-outlier methods (Mcheza, BayeScan) and by a landscape genomics correlative approach (Matsam) definitely strengthens the robustness of the list of loci identified as under selection and possibly involved in adaptation processes, and, in the same time, constitutes a useful cross-validation or double-check [18].

Having said this, it has to be noted that the results of F_ST_-outlier approaches like Mcheza and BayeScan can be biased by factors like the strength, pervasiveness or time frame of the selection processes [45]. Further difficulties in the use of these methods may rise when processing very large datasets produced by high-throughput sequencing technologies. Calculation time will increase dramatically and may become an obstacle for many users. Correlative approaches that do not require the processing of simulated data are much more computation-effective.

Finally, F_ST_-outlier detection methods can be affected by population structure, since demographic processes (e.g. bottlenecks) or introgression can sometimes leave genomic signatures that mimic selection [46]. This complicates the task of disentangling selection from demography [47]–[48]. Novel approaches like the detection of patterns of linkage disequilibrium, as reviewed by Li and colleagues [47], may permit to address this issue once applied to loci identified as possibly under selection by correlative approaches. Indeed, a combined approach able to capitalize on the robust theoretical framework of theoretical population genetics as well as on the incorporation of the effect of landscape spatial heterogeneity would constitute an optimal solution [42].

Opposite to F_ST_-outlier methods which only identify selected loci, correlative approaches as Matsam return both the list of selected markers and of the environmental variables constituting the probable selective pressure. The landscape genomics approach, by providing knowledge about the nature of the selective pressure, is likely to favour the emergence of working hypotheses about the role of the genomic regions surrounding the markers under study, and thus facilitate the discovery of genes possibly involved in specific functional processes; in this specific hypothesis-driven context, it is useful to mention that bivariate models may be elaborated to refine the analysis. An important difference is that, being independent of any theoretical genetic framework and having no constraint like Hardy-Weinberg or Linkage equilibrium assumptions, landscape genomics is not able to discriminate between directional and balancing selection.

### The role of livestock landscape genomics

In spite of the reservations mentioned above, we want to stress here the importance of livestock landscape genomics, in particular in developing countries. In Africa, it is widely recognized that livestock genetic resources - and as a consequence their germplasm and adaptive potential - are now endangered, since local breeds are being gradually substituted by or “improved” through crossing with cosmopolitan breeds. Local breeders decisions are more and more influenced by the need to increase short-term productivity [49], with the consequence of becoming dependent on expensive external inputs, such as veterinary intervention to progressively face increased diseases susceptibility or fertility loss. This leads local breeders to lose their means of sustenance and join shantytowns around big cities, with a reduced level of livelihood and of food security. Landscape genomics can also be used to obtain an empirical evaluation of biodiversity patterns within a geo-environmental context when no direct information is available on livestock diversity levels, and may constitute an opportunity to reverse the trend of farmers' livelihood worsening described above. Indeed, if more attention was paid to local environmental conditions, animals and breeds could be more efficiently selected to increase the overall livestock performance within a specific environment [50]. The underlying idea, in fact, is that the genotype of animals living in a specific habitat will show signatures of selection in those traits significantly associated with habitat characteristics, and that the added effect of human selection will point to genome regions linked to productivity traits [49]. It is, therefore, conceivable that in the near future the concurrent application of landscape genomics and genomic selection will provide flocks tailored for specific environments and with improved production efficiencies [50]. Concerning our study, an important next step will be to locate the AFLP markers most significantly associated to environmental variables on the goat genome, once a complete and reliable assembly will be available, and to relate them to functionally relevant genes [17], [51]. In the next future, the increasing use of goat SNP panels for genotyping purposes will greatly increase the resolution power of the landscape genomics approach and will eliminate mapping problems. Main issues regarding the adoption of SNP panels data for landscape genomic evaluations will be the absence of ascertainment bias and the incorporation of genetic variants typical of local breeds from different agro-climatic areas worldwide.

In developing countries, the conservation of local genetic resources is fundamental to guarantee a sustainable livelihood for over one billion people on the Earth. Livestock landscape genomic approach holds the potential to explore the genomic bases of the adaptation of livestock breeds to specific environmental conditions. Besides adding conservation value to livestock genetic resources and helping in the definition of conservation units [52], in the present-day context of rapid global climatic change, the incorporation of genome-landscape interaction data will permit to develop novel molecular tools useful to i) conserve the adaptive potential during genetic improvement programs of local breeds, and ii) increase adaptability in highly productive industrial breeds.

## Supporting Information

Figure S1 **Geographical position of the farms where the goats have been sampled.** For the correspondence between numbers and breed names, see Table 1.
(TIF)

Figure S2 **Vennes diagram showing the sets of loci possibly under selection as returned by the three methods used.** The significance threshold for Matsam was set to 8.3E-16 (Bonferroni correction included). For Mcheza the confidence level is 99%, while it was set to log10(BF) = 3 for BayeScan. The number of environmental variables identified by Matsam as significantly associated with a locus is given into brackets. Loci written in *italic* are possibly under balancing selection.
(TIF)

Figure S3 **Breed by breed frequency of M16, M86, M89 and M96, the four AFLP markers which have been identified as the most significantly associated to environmental variables.** Breeds are geographically ordered from the left (West) to the right (East). Breed acronyms are explained in Table 1.
(TIF)

File S1 **Lab protocol and primer sequences used for the production of AFLP markers.**
(DOCX)
