# Supplementary figures and images for: Assessing The Spatial Dependence of Adaptive Loci in 43 European and Western Asian Goat Breeds Using AFLP Markers
Source: PLoS One. 2014 Jan 30;9(1):e86668. doi: 10.1371/journal.pone.0086668 (PMC3907386; doi:10.1371/journal.pone.0086668)

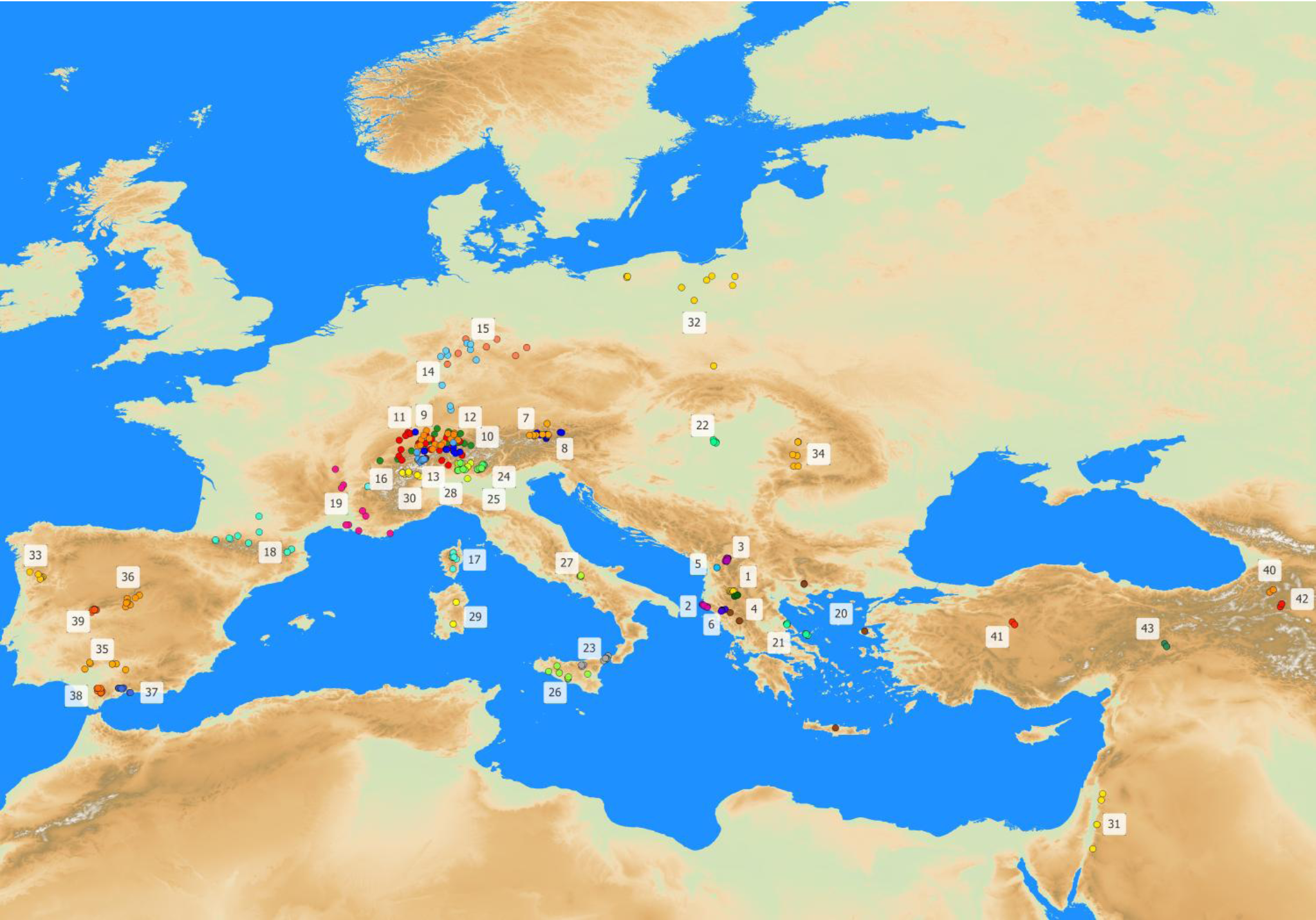

Supplement: Figure S1 — Geographical position of the farms where the goats have been sampled. For the correspondence between numbers and breed names, see Table 1. (TIF) [file pone.0086668.s001.tif]

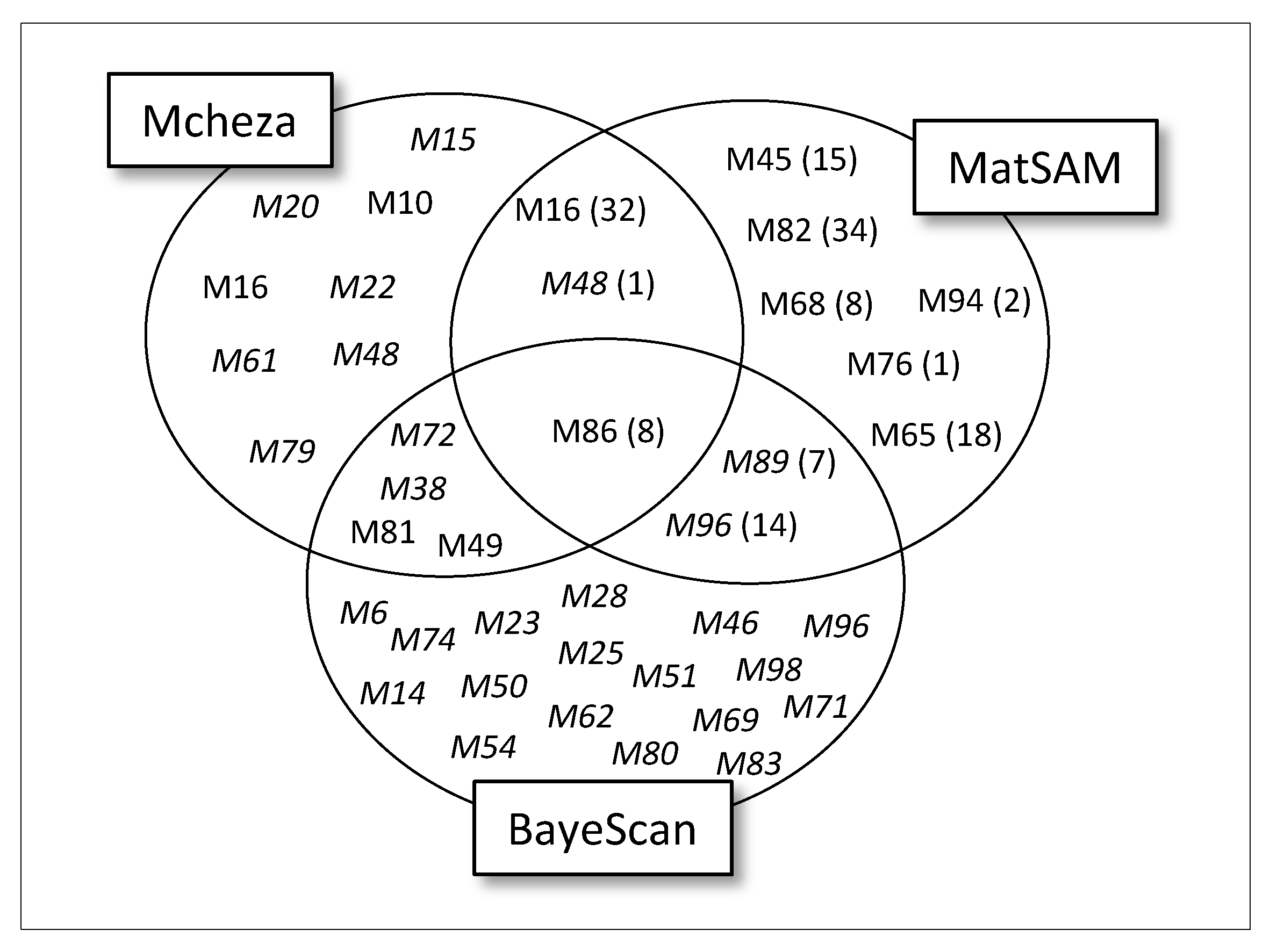

Supplement: Figure S2 — Vennes diagram showing the sets of loci possibly under selection as returned by the three methods used. The significance threshold for Matsam was set to 8.3E-16 (Bonferroni correction included). For Mcheza the confidence level is 99%, while it was set to log10(BF) = 3 for BayeScan. The number of environmental variables identified by Matsam as significantly associated with a locus is given into brackets. Loci written in italic are possibly under balancing selection. (TIF) [file pone.0086668.s002.tif]

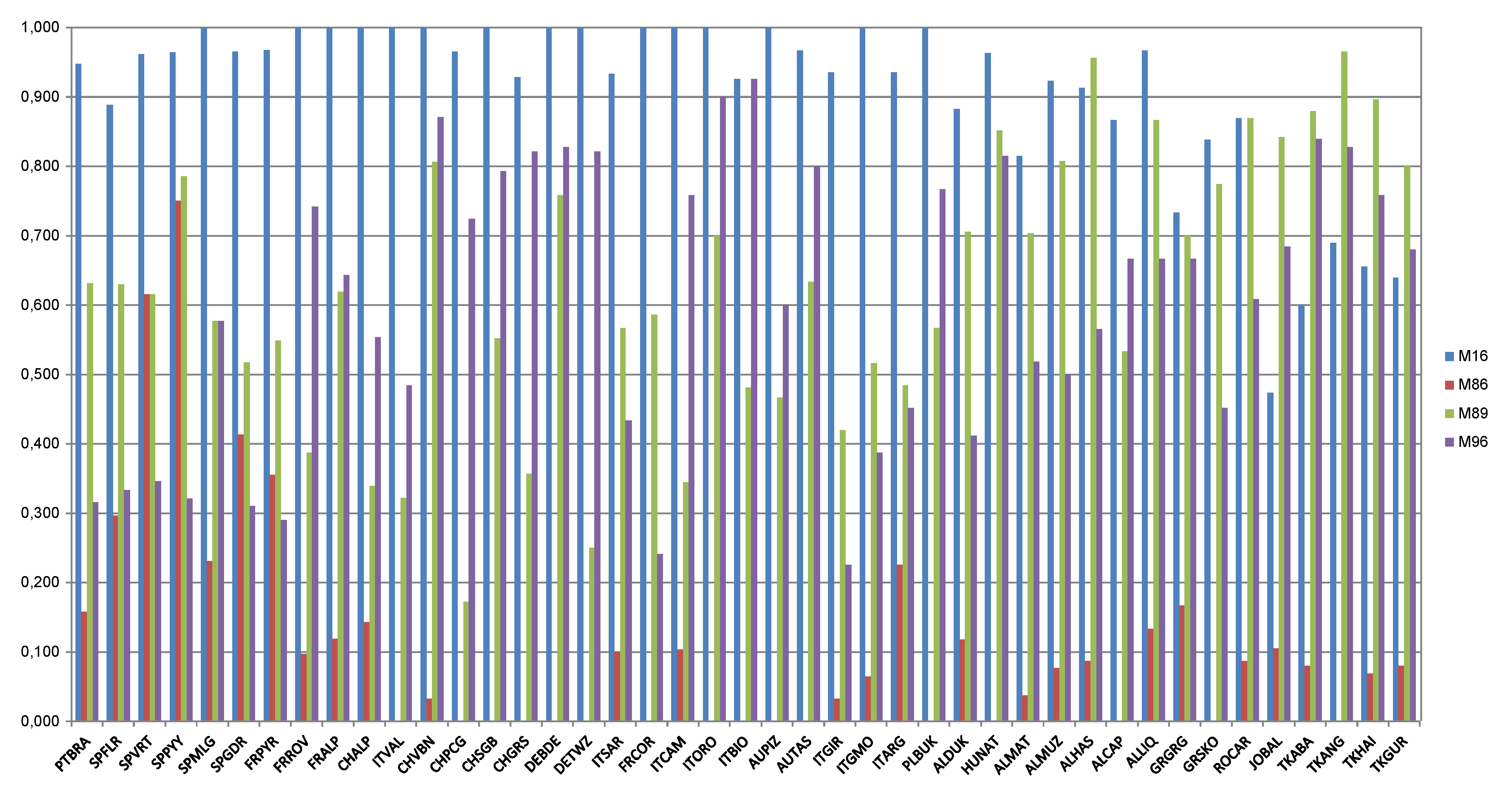

Supplement: Figure S3 — Breed by breed frequency of M16, M86, M89 and M96, the four AFLP markers which have been identified as the most significantly associated to environmental variables. Breeds are geographically ordered from the left (West) to the right (East). Breed acronyms are explained in Table 1. (TIF) [file pone.0086668.s003.tif]
