# Supplementary material for: Assessing The Spatial Dependence of Adaptive Loci in 43 European and Western Asian Goat Breeds Using AFLP Markers
Source: PLoS One. 2014 Jan 30;9(1):e86668. doi: 10.1371/journal.pone.0086668 (PMC3907386; doi:10.1371/journal.pone.0086668)
Supplement: File S1 — Lab protocol and primer sequences used for the production of AFLP markers. (DOCX) [file pone.0086668.s004.docx]

**File S1:**

**AFLP protocol**

To produce *Eco*RI/*Taq*I AFLP markers the following standardized protocol was adopted: 100 ng of DNA was incubated for 1 h at 65°C with 5 U of *Taq*I restriction enzyme (New England Biolabs Inc, Ipswich, MA) in a volume of 25 ul containing: 10 mM Tris-HAc (pH 7.5), 10 mM MgAc, 50 mM KAc, 5 mM dithiothreitol (DTT) and 50 ng/ul bovine serum albumin (BSA).

In the next step, 15 ul of a solution having the same DTT, BSA and ion composition and containing 5 U of *Eco*RI restriction enzyme (New England Biolabs Inc, Ipswich, MA) was added and the resulting 40 ul was incubated at 37°C for 1 h.

To ligate adapters, 10 ul of a solution containing 5 pmol *Eco*RI adapters, 50 pmol *Taq*I adapters, 1 U of T4 DNA ligase (New England Biolabs Inc, Ipswich, MA) and 1 mM ATP, in the same salt, DTT and BSA concentration, was added and the final 50 ul incubated at 37°C for 3 h.

Template DNA was diluted 1:10 before further use.

Pre-amplification reactions were performed by adding 5 ul of diluted template DNA to a PCR reaction mix containing: 1x reaction buffer, 1.5 mM MgCl_2_, 0.2 mM dNTPs, 1 U of Applied Biosystems^®^ AmpliTaq^®^ DNA Polymerase (Life Technologies Ltd, Paisley, UK) and 75 ng each of *Eco*RI and *Taq*I primers carrying one selective nucleotide (primers E01 and T01 in Table S1), in a total volume of 50 ul. Samples were subjected to 30 cycles of (pre)amplification with the following profile: 30 s at 94°C, 1 min at 56° and 1 min at 72°C, followed by 10 min at 72°C for the completion of partial amplifications. The preamplified template was diluted 20-fold and processed further.

To perform the selective amplification step, 5 ul of diluted preamplified template was added to a PCR reaction mix containing 1x reaction buffer, 1.5 mM MgCl_2_, 0.2 mM dNTPs, 0.4 U of Applied Biosystems^®^ AmpliTaq^®^ DNA Polymerase (Life Technologies Ltd, Paisley, UK), 5 ng ^33^P terminally labeled *Eco*RI primer and 30 ng unlabelled *Taq*I primer, both carrying three selective nucleotides each (Table S1), in a total volume of 20 ul.

The following thermal profile, including a total of 36 cycles, was used: 30 s at 94°C for initial

denaturation, 30 s at 65°C as first annealing step and 1 min at 72°C for primer extension. In each

following cycle, the annealing temperature was reduced by 0.7°C down to 56°C and thereafter

kept constant until the completion of PCR run. The touchdown PCR strategy was adopted to ensure high-stringency amplification.

Table S1

Adapters and primers used in AFLP analysis (selective nucleotides in bold).

| *Eco* RI adapters | *Eco* top strand | CTC GTA GAC TGC GTA CC |
| --- | --- | --- |
|  | *Eco* bottom strand | AAT TGG TAC GCA GTC TAC |
| *Taq* I adapters | *Taq* top strand | GAC GAT GAG TCC TGA C |
|  | *Taq* bottom strand | CGG TCA GGA CTC AT |
| *Eco* RI primers | E01 (pre-amplification) | GAC TGC GTA CCA ATT C**A** |
|  | E32 | GAC TGC GTA CCA ATT C**AA C** |
|  | E43 | GAC TGC GTA CCA ATT C**AT A** |
|  | E45 | GAC TGC GTA CCA ATT C**AT G** |
| *Taq* I primers | T01 (pre-amplification) | GAT GAG TCC TGA CCG A**A** |
|  | T32 | GAT GAG TCC TGA CCG A**AA C** |
|  | T33 | GAT GAG TCC TGA CCG A**AA G** |
|  | T38 | GAT GAG TCC TGA CCG A**AC T** |

*Detection and scoring of AFLP markers*

^33^P terminally labeled amplification products were mixed with an equal amount of formamide loading buffer, denatured and separated on a 40 cm 4.5% polyacrylamide sequencing gel, run in TBE buffer (100 mM Tris-HCl, 100 mM Boric acid, 2 mM EDTA) at constant wattage (56 W) for 2 hours.

Following the run, the gel was fixed for 20 min in 10% acetic acid, rinsed with deionized water, dried and autoradiographed on Amersham Hyperfilm MP for 48 h (Amersham International, Little Chalfont, UK). AFLP markers were read visually from autoradiographs in a size range from 50 to 600 bp.
